# Robotic Total Gastrectomy With Intracorporeal Robot-Sewn Anastomosis: A Novel Approach Adopting the Double-Loop Reconstruction Method

**DOI:** 10.1097/MD.0000000000001922

**Published:** 2015-12-11

**Authors:** Amilcare Parisi, Francesco Ricci, Stefano Trastulli, Roberto Cirocchi, Alessandro Gemini, Veronica Grassi, Alessia Corsi, Claudio Renzi, Francesco De Santis, Adolfo Petrina, Daniele Pironi, Vito D’Andrea, Alberto Santoro, Jacopo Desiderio

**Affiliations:** From the Department of Digestive Surgery, St. Mary's Hospital, University of Perugia, Terni (AP, JD, ST, RC, FR, VG); Department of General and Oncologic Surgery, University of Perugia, Perugia (AC, CR, AG, FDS, AP); and Department of Surgical Sciences, Sapienza University of Rome, Rome, Italy (DP, VD, AS).

## Abstract

Gastric cancer constitutes a major health problem. Robotic surgery has been progressively developed in this field. Although the feasibility of robotic procedures has been demonstrated, there are unresolved aspects being debated, including the reproducibility of intracorporeal in place of extracorporeal anastomosis.

Difficulties of traditional laparoscopy have been described and there are well-known advantages of robotic systems, but few articles in literature describe a full robotic execution of the reconstructive phase while others do not give a thorough explanation how this phase was run.

A new reconstructive approach, not yet described in literature, was recently adopted at our Center.

Robotic total gastrectomy with D2 lymphadenectomy and a so-called “double-loop” reconstruction method with intracorporeal robot-sewn anastomosis (Parisi's technique) was performed in all reported cases.

Preoperative, intraoperative, and postoperative data were collected and a technical note was documented.

All tumors were located at the upper third of the stomach, and no conversions or intraoperative complications occurred. Histopathological analysis showed R0 resection obtained in all specimens. Hospital stay was regular in all patients and discharge was recommended starting from the 4th postoperative day. No major postoperative complications or reoperations occurred.

Reconstruction of the digestive tract after total gastrectomy is one of the main areas of surgical research in the treatment of gastric cancer and in the field of minimally invasive surgery.

The double-loop method is a valid simplification of the traditional technique of construction of the Roux-limb that could increase the feasibility and safety in performing a full hand-sewn intracorporeal reconstruction and it appears to fit the characteristics of the robotic system thus obtaining excellent postoperative clinical outcomes.

## Introduction

Minimally invasive surgery (MIS) has been developed over the past 2 decades as a feasible approach for the treatment of gastric cancer.

Nowadays, MIS is generally accepted as an alternative to open surgery in the treatment of early gastric cancer (EGC), whereas for advanced gastric cancer (AGC), the execution of this approach can be considered if an adequate lymph-node dissection is guaranteed to the patient.

More recently, technological advancements with the spread of robotic systems have improved intracorporeal movements and visualization with a 3d vision.

Nevertheless, there are still few studies on this field and many aspects are controversial.

Robotic technology could overcome the limitations of traditional laparoscopy and most reports emphasize the easier handling, in particular in the dissection phase, even if advantages have not been proven by randomized controlled trials.

In the current literature, an interesting aspect is that the way to perform the reconstructive phase is not properly discussed and it seems to take a second place, even more regarding the total gastrectomy.

However, surgeons well know that the anastomosis execution method has the most important impact on perioperative outcomes, such as hospital stay and surgical complications.

Studies do not explain how this phase of the intervention is run or study groups were made up of mixed procedures without subgroup analysis.

In particular, in the field of the total gastrectomy, different technical possibilities were described, but only 2 studies^1,2^ highlighted the potentiality of the robotic system in performing a complete robot-sewn anastomosis. On the contrary, all other studies reported extracorporeal or mechanical stapler methods.^3–18^

The advent of robotic surgery, with the aid of microsurgical instruments with 7 degrees of freedom, has provided a noticeable boost to the possibility of performing completely intracorporeal sutures.

This study aims to describe the robotic total gastrectomy with a double-loop robot-sewn intracorporeal reconstruction, conceived at our Institute and not described in previous articles.

## MATERIALS AND METHODS

Between May 2014 and July 2015, 22 patients underwent the robotic double-loop reconstruction method (called Parisi technique) after performing a robotic total gastrectomy with extended lymphadenectomy for histologically proven gastric adenocarcinoma.

Preoperative, intraoperative, and postoperative data were collected in a prospective database. Patients’ demographics are summarized in Table 1.

**TABLE 1:**
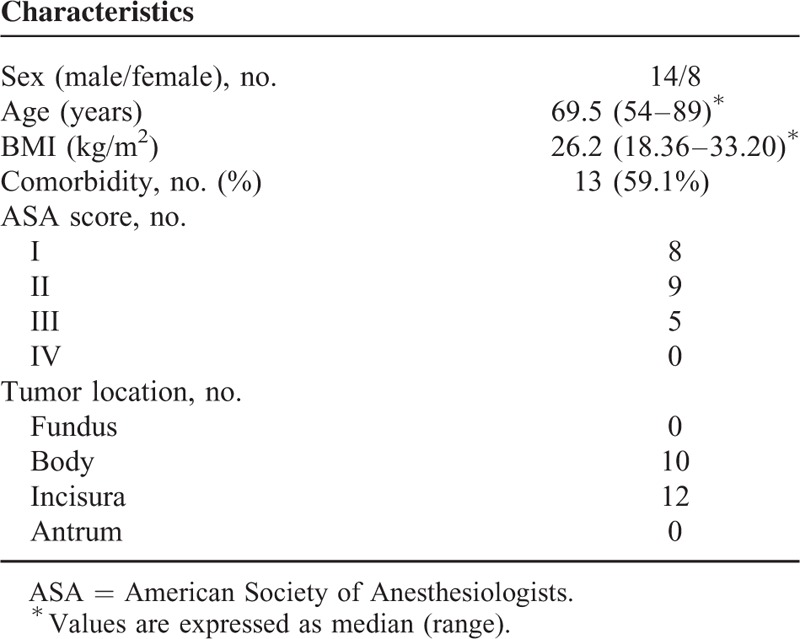
Characteristics of Enrolled Patients

Procedures and lymphadenectomies were carried out according to the Japanese Classification of Gastric Cancer (JCGC, third edition).^19^ The pathologic stage classification of the tumor was worked out according to the AJCC Cancer Staging Manual Seven Edition.^20^

Inclusion criteria of patients’ enrollment for this intervention were histologically proven gastric cancer, preoperative staging work-up performed by upper endoscopy and/or endoscopic ultrasound, computed tomographic (CT) scan, both EGC and AGC, and patients treated with curative intent in accordance to international guidelines.

Exclusion criteria were locally advanced, tumor-infiltrating neighboring organs, distant metastases, Krukenberg tumors, patients with a high operative risk as defined by the American Society of Anesthesiologists^21^ score ≥4, history of gastric surgery, remnant gastric cancer, synchronous other major abdominal surgery, synchronous malignancy in other organs, and palliative surgery cases.

Preoperative, intraoperative, postoperative data were collected.

The analyzed outcomes included patient characteristics and tumor, overall operative time (from the start of pneumoperitoneum until suture of all surgical incisions), conversion to open surgery, site of mini-laparotomy for specimen extraction, intraoperative blood loss (IBL), intraoperative complications, time to first bowel movements, time to liquid and solid intake, postoperative complications (from the end of surgery until discharge), mortality, hospital stay (starting from the day of surgery till discharge), 30-day complications (after discharge), and histopathological features.

A moving average chart was performed on the operative time, while a cumulative sum (CUSUM) plot was used to assess surgical success. Surgical failure was defined as conversion to open surgery; operative time ≥mean + 1SD (274.3 + 54.5 min); failure to harvest an adequate number of lymph nodes (≤15 nodes); resection margin involvement; perioperative major complications (mortality, bleeding, pancreatitis, leak, stenosis); hospital stay ≥mean + 1SD (6.33 + 3.85 days).

### Statistical Analysis

SPSS statistics 20 was used to carry out this statistical analysis. The dichotomous variables will be expressed as numbers and percentages, while continuous variables will be expressed as mean and standard deviation^22^ or median and interquartile range (minimum and maximum values).

Paired *t* test was used for testing the mean difference between paired observations.

We considered an α = 0.05 for the level of significance and regarded *P* values <0.05 as statistically significant.

Minitab 17.1.0 was used for the cumulative-sum control chart and the moving average control chart.

## SURGICAL TECHNIQUE

### Patient Preparation

Patients were evaluated with outpatient scheduled visits before surgery and studied with upper endoscopy and biopsy, endoscopic ultrasound, and CT scan.

All patients gave informed consent during the first interview in which characteristics of surgery were explained.

All patients were given antibiotic prophylaxis with cefazolin, administered pre-operatively and continued for 48 hours at a dose of 1 g every 6 hours.

Antithrombotic prophylaxis consisted of an intermittent compression device for the legs held in place during surgery and enoxaparin sodium injections in the early postoperative period.

### Patient Position

**FIGURE 1:**
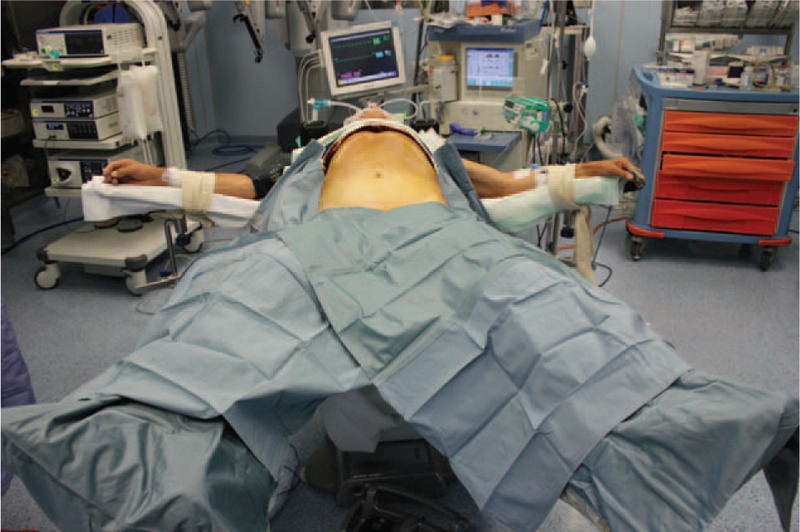
Patient preparation.

After induction of general anesthesia, the patient receives a nasogastric tube and urinary catheter and is placed supine with both legs in abduction (Fig. 1). Arms are also abducted at 90°. The table is inclined in reverse Trendelenburg at approximately 15°. Monitors are placed above patient's head.

### Port Placement

The pneumoperitoneum is established by Veress needle and the insufflator is set to a pressure of 12 mm Hg.

**FIGURE 2:**
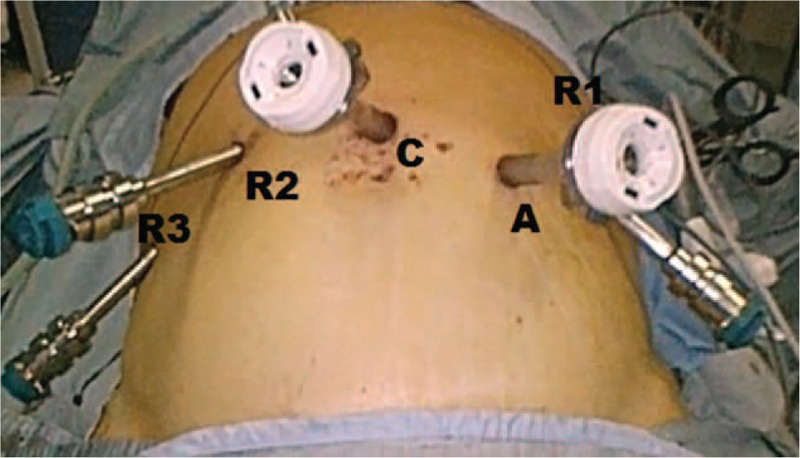
Trocars disposition.

Figure 2 shows trocars position. One 12 mm trocar (C) is placed on the midline just above the navel for optical devices. Three 8 mm robotic trocars are positioned, under camera visualization, as follows: one in the left upper quadrant along the right midclavicular line (R1), the other one in the right upper quadrant along the left midclavicular line (R2), and the third along the right anterior axillary line (R3).

A 12 mm extra-port (A) is inserted and placed between the robotic port R1 and the camera port. It is used by the assistant surgeon in the various surgical phases to introduce the aspirator, mechanical stapler, and stitches.

### Laparoscopic Phase and Coloepiploic Mobilization

In a first phase, a laparoscopic exploration of the abdominal cavity is performed to exclude the presence of metastases of the parenchymatous organs and the peritoneal cavity. The precise location of the tumor and its local extension is also accessed.

Then, a first mobilization phase is performed by laparoscopy to evaluate the relationship between the tumor and the nearby organs, especially the posterior structures.

Thus, a complete coloepiploic mobilization is performed using the harmonic scalpel from right to left.

This phase can be conducted after the robotic docking or by laparoscopy depending on patient's characteristics and surgeon's preference.

The surgeon proceeds with the dissection, opening the epiploon retrocavity. The transverse colon is turned down by an assistant and the detachment is carried out toward the lower pole of the spleen.

The lymph node station no. 4d, along the second branch and distal part of the right gastroepiploic artery, is removed.

The origin of the left gastroepiploic artery is found at the distal end of the pancreatic tail.

This section allows the surgeon to remove the lymph nodes of the station no. 4sb.

After reaching the splenic hilum, the detachment is continued above so as to dissect the short vessels connecting the gastric fundus with the spleen.

The release of the gastric fundus thus enables to remove lymph nodes of the station no. 4sa.

### Robotic Docking

**FIGURE 3:**
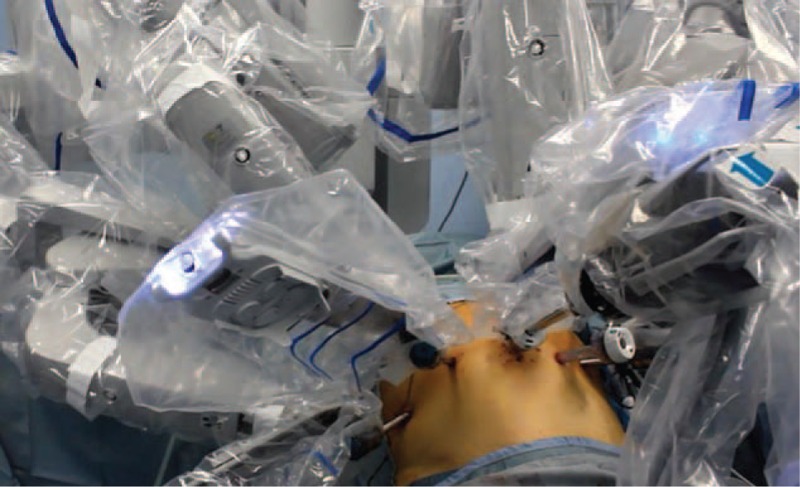
Robotic docking.

The 4-arm da Vinci Surgical System is docked to the operative table above the patient's head and the operative arms are connected to the ports (Fig. 3). The 30° robotic camera is inserted through the supra-umbilical port. The surgeon at the console controls the robotic arm no. 1 with the right master and the 2 left arms (robotic arms no. 2 and no. 3) switching between both these arms depending on the steps of the procedure.

The working arms carry a cautery hook on the first robotic arm and a fenestrated bipolar forceps on the second robotic arm. Also, a double fenestrated grasper is positioned on robotic arm no. 3 to help with retraction and used to lift the liver. The 12 mm assistant port is used to assist the surgeon in several steps of the procedure and for the introduction of aspiration/irrigation device, clip applier, linear stapler, sponges, and sutures.

### Ligation of the Right Gastroepiploic Artery

The Grasper of the third robotic arm is placed on the rear face of the stomach exposing the duodenal region.

The superior right colic vein is identified in order to find the gastrocolic trunk of Henle. The latter is dissected and the right gastroepiploic vein is sectioned at its origin.

Following the anterior face of the pancreas, the right gastroepiploic artery is reached and tied, applying hem-o-lock, at its origin from the gastroduodenal artery.

All the cellular tissue that surrounds this artery is removed with the specimen and contains the lymph nodes of station no. 6. Thus, the pylorus and the first portion of the duodenum are fully released.

### Ligation of the Right Gastric Artery and Section of the Duodenum

Then, the stomach is overturned downward on the left and the third robotic arm is used to lift the left hepatic lobe ensuring the necessary space for the dissection performed with the cautery hook.

The lesser omentum is cut close to the liver, from pars flaccida to the hepatic pedicle.

This dissection allows the surgeon to remove the station no. 3, representing the lymph nodes located near the small curve along the lower branch of the left gastric artery up to the right gastric artery.

The proper hepatic artery is prepared from the top downwards, so as to identify the right gastric artery. The latter is sectioned between hem-o-lock at its origin, removing the adipose tissue containing station no. 5.

In this way, the release of the first part of the duodenum is completed.

**FIGURE 4:**
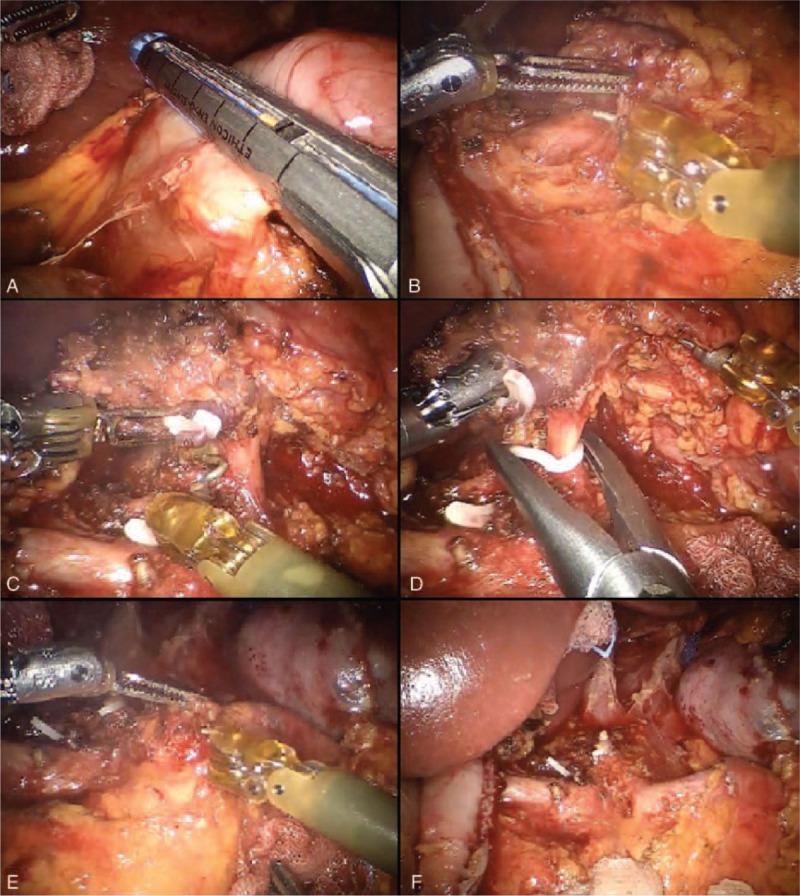
Lymphadenectomy of major vessels.

The assistant introduces, through the A port, an articulated linear mechanical stapler with a visceral cartridge, placing and firing it 1 cm downstream from the pylorus (Fig. 4A).

### Lymphadenectomy of Major Vessels

Now, the surgical specimen is brought upward and to the left, substained by the third robotic arm, thus exposing the celiac trunk region.

The cautery hook permits a gentle dissection, while the bipolar forceps can contextually coagulate, thus baring the adventitia of vessels.

The lymph node dissection starts from the hepatic hilum toward the celiac trunk (Fig. 4B). The adipose tissue at the upper left portion of the pedicle and proper hepatic artery is removed (station no. 12a).

The dissection continues at the level of the left portion of the hepato-duodenal ligament and the upper edge of the pancreas.

The common hepatic artery is stripped of the cellular tissue from the origin of the gastro-duodenal artery to the celiac trunk (station no. 8).

Using microsurgical instruments easily, the dissection is gradually pushed along the vessels.

The dissection continues to the left, at the level of the celiac trunk, until its origin from the aorta and then to its splenic and left gastric branches of division, thus removing the station no. 9 (Fig. 4C).

Once the celiac trunk is released, the left gastric artery can be easily controlled and tied at its origin between hem-o-lock, so as to remove the station no. 7 (Fig. 4D).

At this time, the left gastric vein is also isolated and sectioned.

The course of the splenic artery is identified and the dissection prolonged to include the proximal splenic artery lymph nodes (station no. 11p) from its origin to halfway between its origin and the pancreatic tail end (Fig. 4E).

The lymphadenectomy of the splenic artery can be concluded at this point or continued toward the distal stations, according to the characteristics of the tumor (Fig. 4F).

In the latter case, the lymphatic tissue is removed from the distal splenic artery (station no. 11d) and the splenic hilum (station no. 10), with or without spleen preservation.

At this point, the surgeon faces the abdominal esophagus. Stomach and omentum are overturned down.

The dissection of the pars flaccida continues close to the liver, from the bottom upwards, in the direction of the diaphragm.

The pars condensa is also tied, carrying up the dissection to the right pillar.

The pre-esophageal peritoneum is opened reaching the left section of the gastro-diaphragmatic ligament.

The access to the right pillar releases the right edge of the esophagus and allows the surgeon to remove all lymph nodes of station no. 1.

The surgical specimen is moved to the left and the dissection of the right posterior surface of the cardia is performed. Then, the stomach is brought to the right to dissect the left pillar and remove the adipose tissue on the left margin of the cardia, representing the station no. 2. The anterior and posterior branches of the vagus nerve are also sectioned.

### Section of the Esophagus

The esophagus is prepared for a length of 3 to 5 cm so as to perform the anastomosis. A needle holder is positioned in place of the cautery hook on robotic arm no. 1.

**FIGURE 5:**
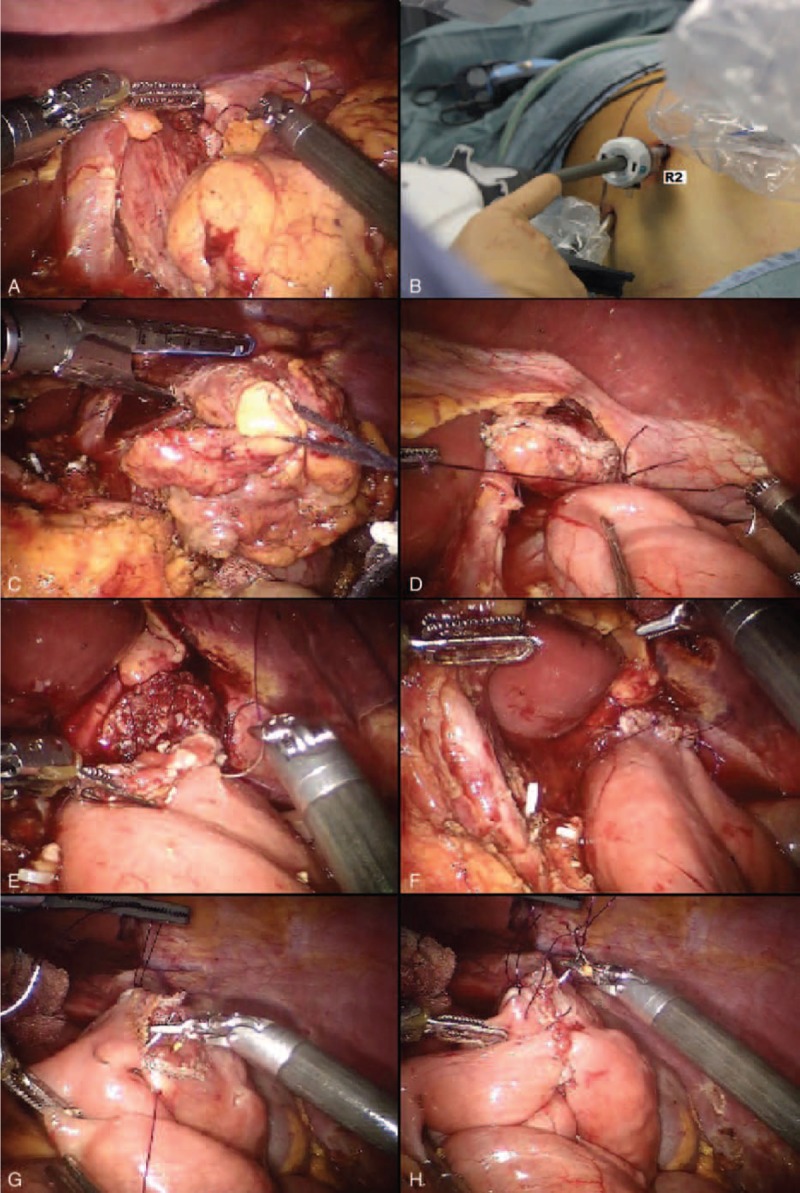
The double-loop reconstruction method (Parisi technique).

Two traction stitches, using Vicryl 2/0, are placed to fix the esophagus to the diaphragm pillars (Fig. 5A). The surgeon chooses an upstream area from the ideal margin of section, so as to ensure the smooth presentation of remaining esophagus during the anastomosis without having the fear of its retraction in the chest.

The assistant undocks the robotic arm no. 2 and replaces the 8 mm robotic trocar with a 12 mm trocar (Fig. 5B), thus permitting the introduction of the mechanical stapler with a correct angle (Fig. 5C).

The esophageal section is performed considering an adequate distance from the tumor.

The robotic arm no. 2 is redocked and armed with the fenestrated bipolar forceps.

So, the restoration of the digestive continuity is run.

### Double-Loop Reconstruction Method (Parisi Technique)

The needle holder is placed on the robotic arm no. 1. The transverse colon is overturned superiorly and the surgeon at the console moves the 3D vision to the submesocolic compartment detecting the angle of Treitz.

From this point of landmark, the intestinal loops are unwound so as to overtake the transverse colon, reaching the sectioned esophagus.

The selected intestinal segment must be free of tension or torsion.

In this maneuver, the side that comes from the Treitz is kept separate from the one that goes to the alimentary tract.

The assistant introduces a stitch with a thread in Vicryl 2/0 of 12 cm length, previously measured. The small intestine is then joined to the esophagus through 2 stitches that pair the esophagus angles to the selected loop so as to find the biliary side on the esophagus left and the alimentary side on the right (Fig. 5D).

They also define the anterior and posterior planes and their thread can be used to adjust tractions.

This represents the first loop.

The sutures are placed using the needle holder on the robotic arm no. 1 and the bipolar forceps on the robotic arm no. 2.

Now, the surgeon can perform an end-to-side esophagojejunal robot-sewn anastomosis.

Initially, the suture is carried on the posterior margin.

A first posterior layer is performed with interrupted stitches taking the jejunal serosal and the esophagus muscle fibers. A measured 12 cm stitch of Vicryl 2/0 is used for each passage.

The small intestine is then opened as well as the end of the esophagus, previously closed by the shot of the stapler.

The second posterior layer is performed by a continuous suture from an angle to the opposite one, taking the entire thickness of the intestinal and esophageal wall (Fig. 5E). In this case, 3/0 PDS is used with a thread measured up to 18 cm.

The jejunum and the esophagus are now well-matched and the suture continues on the anterior plane.

A second continuous suture joins the posterior one at the anastomosis angles.

Then, the anterior plane is finished with interrupted stitches covering the previous layer (Fig. 5F).

At this point, the route of the alimentary limb is followed up to reach a distance of about 30 to 40 cm from the esophago-jejunal anastomosis.

In this way, the second loop is identified. It is carried upward, avoiding intestinal twisting, and placed close to the first anastomosis.

Then, the chosen intestinal segment is joined to the biliary limb with 2 sero-serosal fixation stitches (Vicryl 2/0), defining the jejuno-jejunal anastomosis (Fig. 5G), but so as to leave no more than 2 cm between the 2 anastomoses.

The assistant fires the stapler and then the opening is closed with a first running layer in PDS 3/0 (Fig. 5H) and a second layer of interrupted stitches in Vicryl 2/0.

**FIGURE 6:**
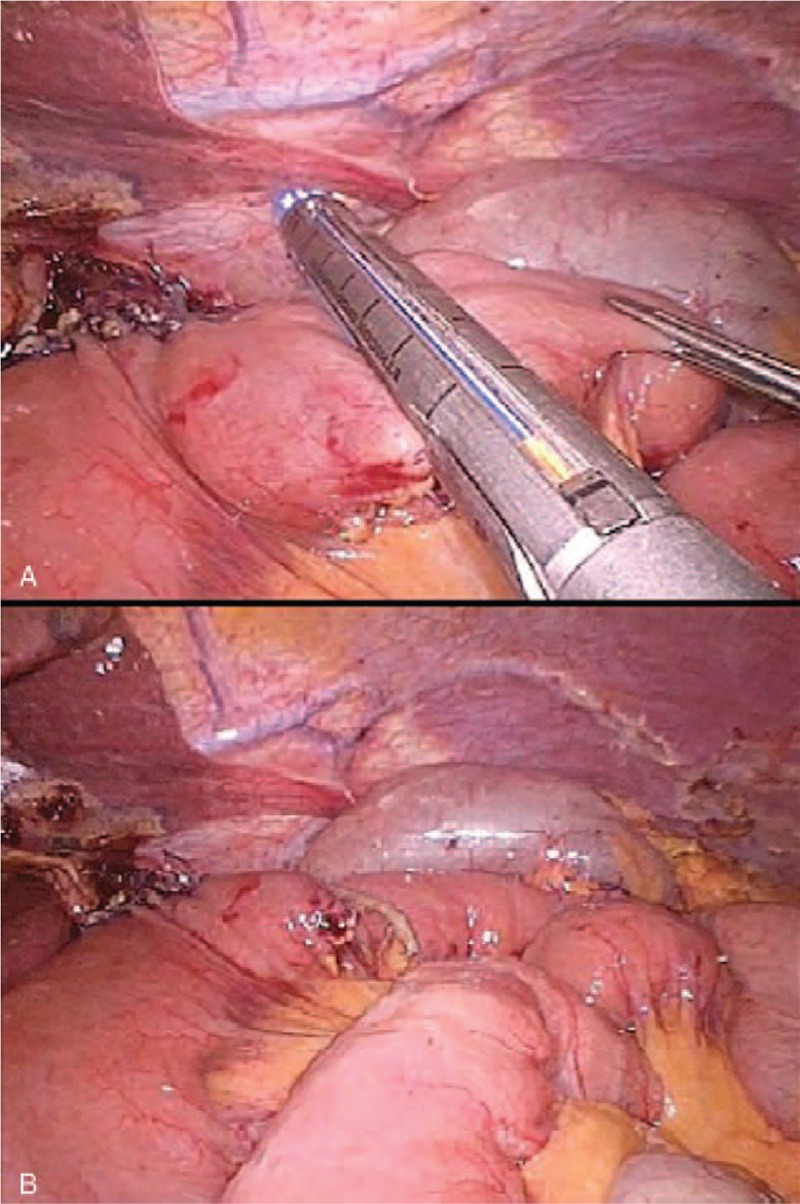
Interruption of continuity between the 2 anastomoses by firing the linear stapler.

The last step of the intervention is the interruption of continuity between the 2 anastomoses to create the Roux-en-Y by firing the linear stapler (Fig. 6A). In this way, after division, the “cul de sac” is minimal (Fig. 6B). Finally, the patency and the tightness of the E-J anastomosis can be tested with methylene blue.

**FIGURE 7:**
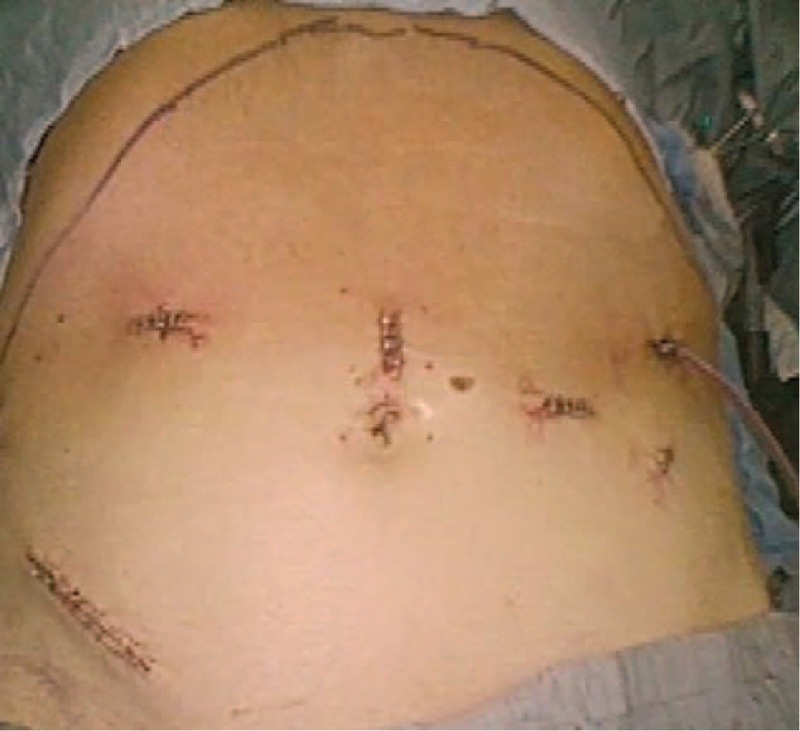
Final abdominal view.

After hemostasis control, a suction drain is positioned close to the esophagus-jejunal anastomosis, while the naso-enteric tube is not placed. At the end, the mini-incisions are sutured (Fig. 7).

## RESULTS

Twenty-two patients (14 males, 8 females; median age: 69.5 years, range 54–89 years) underwent the procedure.

The baseline characteristics of the subjects enrolled in this study are summarized in Table 1.

**FIGURE 9:**
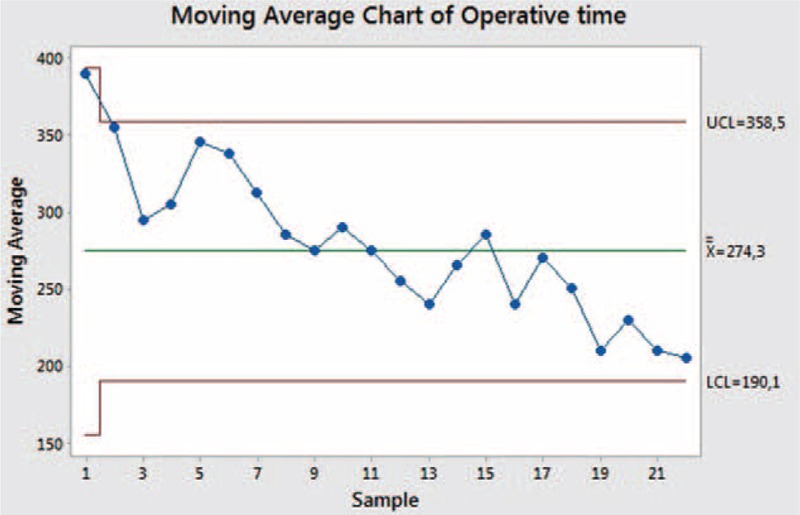
Moving average chart of overall operative time.

The data relating to surgery are summarized in Table 2. Median operative time was 270 min (220–390 min); no procedure was converted. Median IBL was 200 mL (50–450 mL). The moving average control chart, based on the overall operative time, is shown in Figure 9.

**TABLE 2:**
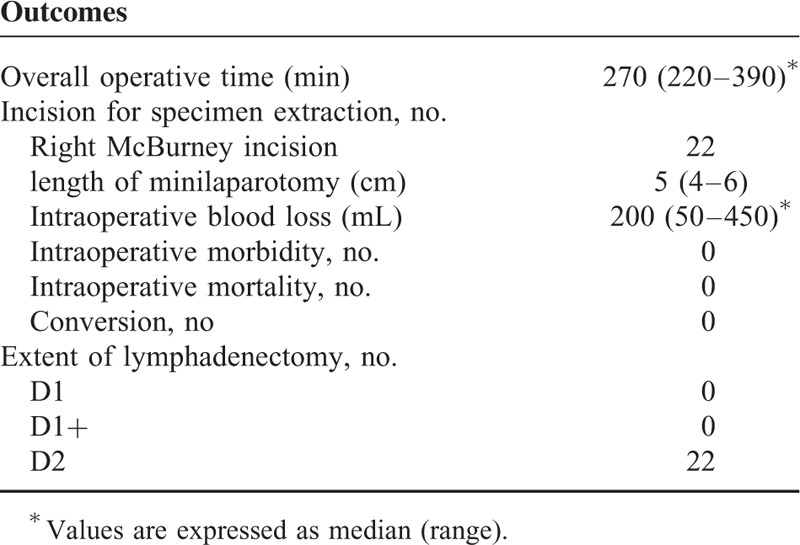
Operative Results

Liquid diet was resumed on the second postoperative day (range: 2–5 days) after a routine postoperative contrast swallow. Median hospital stay was 5.5 days (range: 4–17 days). Other data related to hospitalization are summarized in Table 3.

**TABLE 3:**
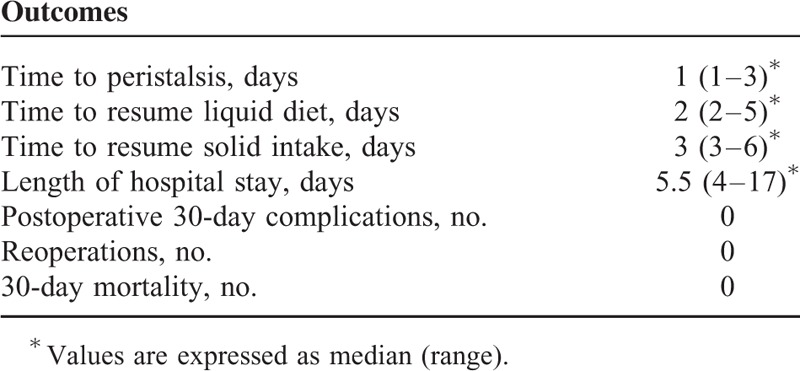
Clinical Outcomes During Hospitalization and Complications

The major complication rate observed in the postoperative period was 0%. Thirty-day mortality was 0%.

Histopathology on the surgical specimen (Table 4) revealed resection margins negative in all cases thus achieving the oncological radicality in all patients. The mean number of harvested lymph nodes was 19.2 ± 5.33 nodes, and the median proximal resection margin was 40 mm (33–70 mm).

**TABLE 4:**
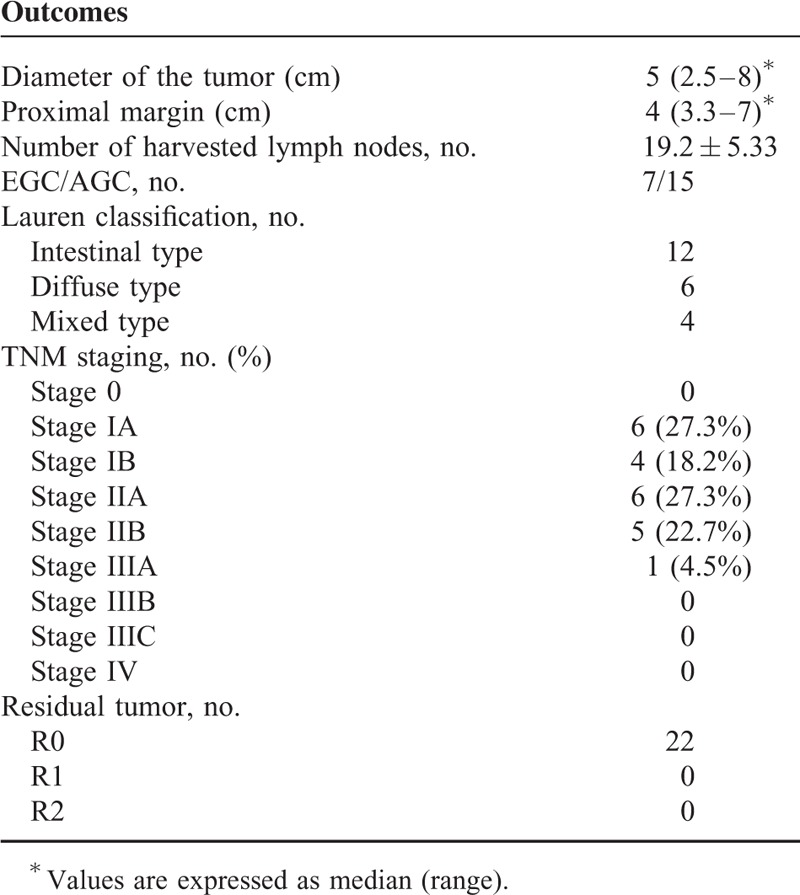
Histopathological Data

**FIGURE 8:**
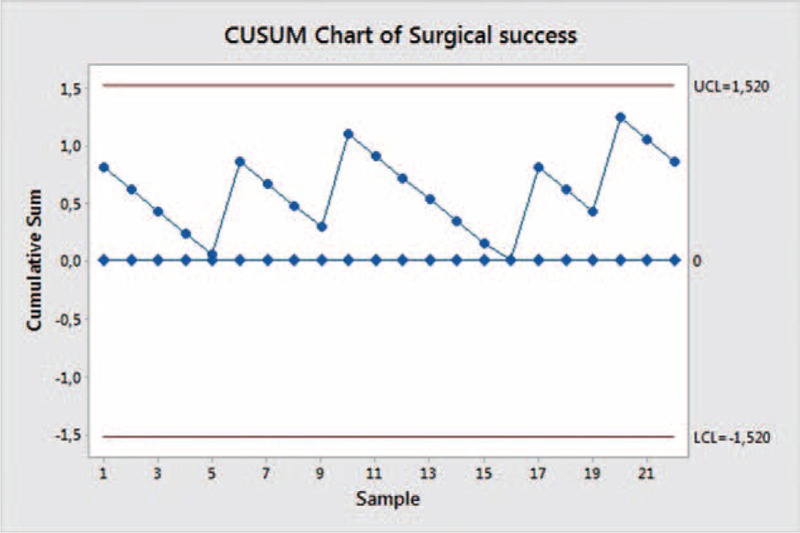
CUSUM chart of surgical success.

The CUSUM analysis (Fig. 8) of the surgical success is reported in Figure 8. The points appear to vary around the center line and are within the control limits. The variability of the procedures appears to be stable.

**FIGURE 10:**
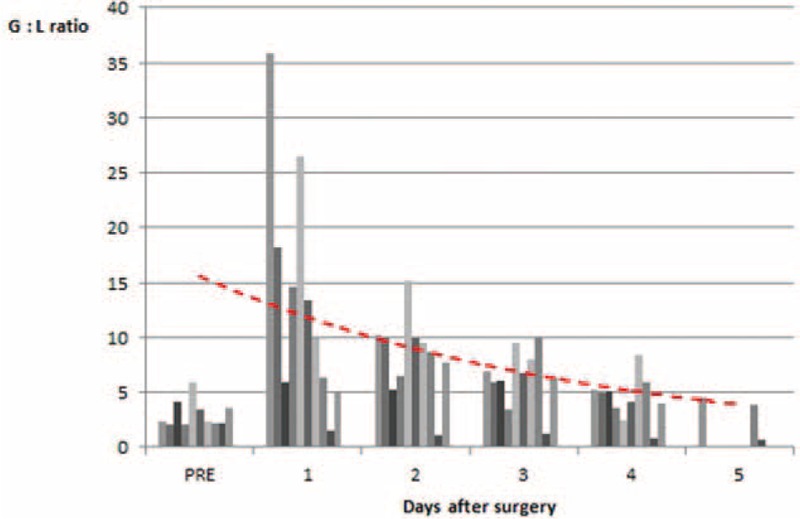
Trend of surgical stress during hospitalization.

Figure 10 graphically shows the G:L ratio trend. The surgical stress was evaluated by comparing the pre-operative to the postoperative values.^11,23^ Particularly, the difference in the G: L ratio between the values detected in the 4th postoperative day and the preoperative ones, resulted not statistically significant. (Table 5).

**TABLE 5:**
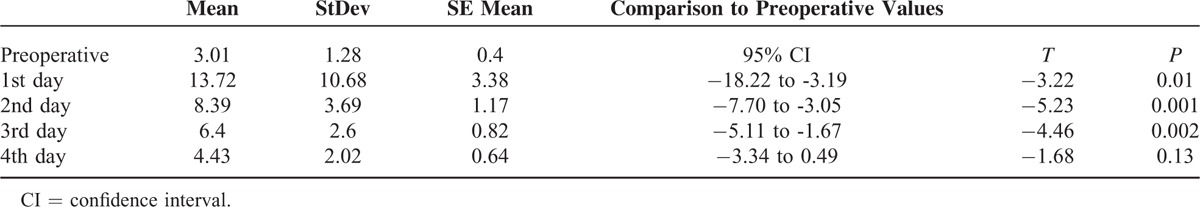
Analysis of Surgical Stress Comparing Preoperative and Postoperative Granulocyte to Lymphocyte Ratio

## DISCUSSION

Gastric cancer is a major worldwide challenge, running rampant in some regions and overall representing the fourth most common cancer.

This disease requires a multidisciplinary context and dedicated institutes, where surgery plays the main role.

Many current areas of research seek to identify the best treatment strategies. Thanks to our continuously evolving technology, MIS has become of increasing interest.

Kitano et al^24^ performed the first laparoscopic-assisted gastrectomy in 1994, and Hashizume and Sugimachi^25^ first used the robotic approach in 2003. These and other surgeons at different centers have published articles on their experiences to define the role of minimally invasive approaches for gastric cancer.^26,27^

However, the current evidence is far from able to prove that these procedures should become a common surgical practice. The current guidelines^28–30^ describe laparoscopy as a possible alternative to open surgery for EGC, while robotic surgery possesses intrinsic technological advantages, but researchers have not verified these advantages through studies with an appropriate level of evidence.

Research in this field, in particular, seeks to assess the effects on perioperative outcomes along with the quality of the patient's life, while still respecting oncological principles. The increasing attention researchers have paid to these approaches unfortunately comes up against the limited data currently available. Thus, many issues have become the subject of debate.

Robotic systems aim to overcome the limits of traditional laparoscopy through 3-dimensional vision, articulated instruments, and the absence of tremor, which result in greater dexterity and precision in the movements of dissection and suturing. These are key elements when performing an extended lymphadenectomy for gastric cancer and a complex and gentle reconstruction to restore digestive continuity. This advanced surgery is also feasible by traditional laparoscopy, but only after a long learning curve that some recent that some recent studies suggest to be shorter with the help of the robot.^31^

Researchers are currently debating several points,^32^ first, the need to perform an adequate lymphadenectomy with minimally invasive techniques. With laparoscopy, surgeons can feasibly remove at least 15 lymph nodes,^26^ but research has shown a statistically significant difference in favor of the open approach regarding the total number of retrieved lymph nodes.

Robotic surgery could overcome the difficulties of traditional laparoscopy and allow a better D2 dissection. Clearly, this high technology possesses intrinsic advantages, but researchers have not yet proven and verified them.

Nevertheless, only 4 studies^3,14,16,33^ have made a comparison with the open approach, and only 3 studies show a statistically significant difference versus laparoscopy.^3,13,16^

IBL correlates with postoperative recovery and cancer cell dissemination. Most studies reported favorable results for MIS versus open surgery. The meta-analysis by Vinuela et al^26^ clearly confirmed this with regard to laparoscopic surgery. Generally, researchers have reported some advantages of robotic gastrectomy over laparoscopic or open surgery in reducing perioperative bleeding.^10,13^ However, several studies have also reported conflicting results.^4,34^

Researchers found a connection between laparoscopy and a significant reduction in overall complications, medical complications, and minor surgical complications as compared with open surgery. They observed that major surgical complications occurred in comparable numbers for the 2 groups. The reduced invasiveness of the laparoscopic technique can explain the decrease in medical and minor surgical complications.^26^ In contrast, the largest randomized controlled trial that the Korean Laparoscopic Gastrointestinal Surgery Study Group performed found no significant difference between the 2 groups in overall complications.^35^

Researchers have obtained inconsistent findings in studies on robotic surgery in terms of demonstrating significant differences compared with laparoscopy.^4,11,36^ However, a careful analysis is limited by the extreme heterogeneity of the surgical techniques adopted.

MIS has demonstrated relevant advantages over open surgery with regard to postoperative hospital stay, despite the extreme heterogeneity among studies.^27^

Some evidence^5,33^ has indicated that patients who underwent robotic gastrectomy could be discharged at an earlier date than patients who underwent open or laparoscopic gastrectomy. However, the low number of studies in this area and the high heterogeneity weaken this conclusion.

New studies must add to the literature and help to conclusively define the best surgical techniques. As Hiki et al^37^ observed, manually handling organs during gastrectomy is an important contributor to the inflammatory response after surgery. Theoretically, the smaller robot instruments may cause less inflammation than the instruments used in other approaches. Thus, postoperative bowel recovery in the robotic group may occur sooner.

Together with the extent of lymphadenectomy, restoration of the digestive tract remains one of the main areas of surgical research and now involves the role of laparoscopic and robotic surgery.

In literature, only 19 studies^1–18,38^ of authors belonging to 14 Institutions reported a total gastrectomy performed with a robot-assisted approach.

Eleven studies are comparative^3–5,8,10–14,16,18^; the remnant are case series and personal experiences.^1,2,6,7,9,15,17,38^ Only 3 studies (Son et al^4^, Yoon et al^8^, Jiang et al^2^) have focused specifically on total gastrectomy, and others reported data and information of surgical interventions, but their analysis included different resection extents.

To date, scientific studies show an overall of 385 interventions of robotic-assisted total gastrectomy.

A thorough analysis shows that in all studies, the robotic assistance was used for lymphadenectomy and stomach mobilization, while regard to the reconstructive time, there is a great variety of solutions adopted and heterogeneity of data.

In fact, only 10 studies^1,2,9–11,14,15,17,18,38^ reported, in a comprehensive manner, the use of the robotic system for performing intracorporeal anastomosis.

Four studies^4–6,16^ in the technical description underline that the surgical team prefers to perform the reconstructive phase by laparoscopy.

Another element that stands out from the analysis is that only 5 studies^4,7,8,13,18^ reported data on the patient and tumor characteristics or on the operative and clinical results.

In particular, this is caused by the absence of subgroup analyzes in articles involving different types of gastrectomies (subtotal and total gastrectomies).

So, you can divide the reconstructive phase into 2 major categories on the basis of the adopted approach: the execution of extracorporeal versus intracorporeal anastomosis.

The latter, in turn, can be performed by laparoscopic assistance or continuing the use of the robotic system.

However, the problem is how to perform the robotic esophago-jejunal anastomosis.

The literature has reported the execution of mechanical anastomosis in most cases, especially with circular staplers through performing a manual purse-string around the anvil.^14,15^ Other solutions described the use of the Orvil^17^ or the Overlap technique.^17,18^

Instead, only 3 authors reported intracorporeal sutures with a completely robotic-sewn anastomosis.^1,10,38^ Among these, only Liu et al^1^ and Jiang et al^2^ provided a detailed description of this procedure.

The advantage of hand-sewn anastomoses is to prevent stricture and encasement; alternatively, this can be solved by using a larger size circular or linear stapler. Regarding this issue, to date, there are no studies that highlight significant differences between the 2 approaches.

In our series, we propose a new robotic approach for the reconstruction phase after total gastrectomy. The Parisi technique is a double-loop reconstruction method with intracorporeal robot-sewn anastomoses.

**FIGURE 11:**
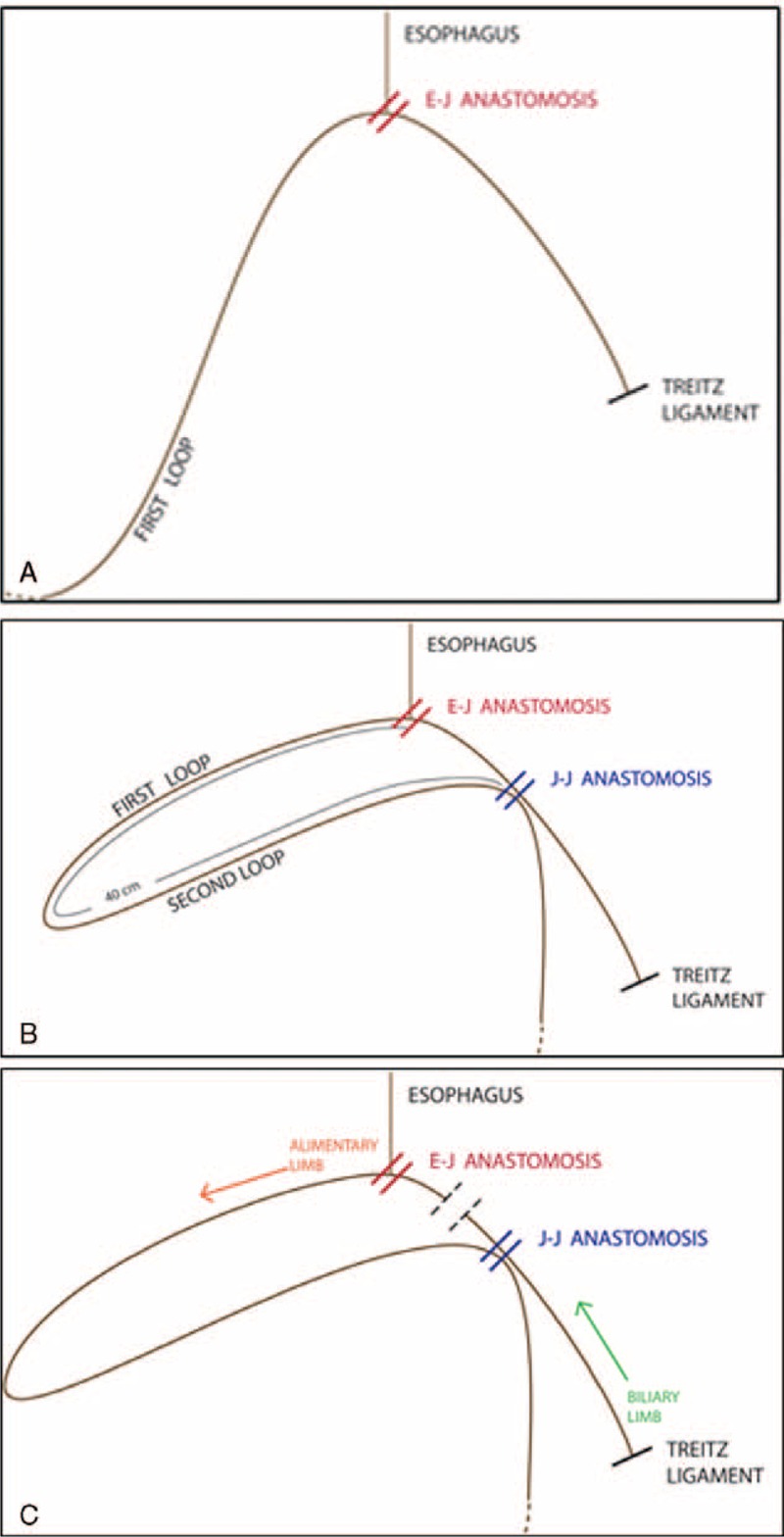
Schematic representation of the Parisi technique during reconstruction. (A) The first loop is made by choosing a jejunal limb that is anastomosed with the esophagus. (B) A second loop is identified downstream to perform the jejuno-jejunal anastomosis. (C) The two anastomoses are divided by simply firing the mechanical stapler, converting the double loop in an antecolic Roux en-Y procedure.

The first loop (Figure 11 A) is made by choosing a jejunal limb that is moved antecolic to the esophagus being careful to be tension free from the Tritz angle. The E-J anastomosis is performed between the first loop and the esophagus. Then, a second loop (Figure 11 B) is identified downstream and is brought close to the E-J anastomosis on its left side. The J-J anastomosis is performed between the second loop and the biliary limb.

At the end of the procedure, just with a single fire of the mechanical stapler, the 2 anastomoses can be divided (Figure 11 C). In this way, after division, the “cul de sac” is minimal.

This procedure represents a variant of the traditional Roux-en-Y reconstruction and was planned to fit the robotic system and to overcome the difficulties that may be encountered in a full intracorporeal reconstruction after total gastrectomy.

Several advantages can be highlighted, which are as follows.

The surgeon can easily adjust the tension of the 2 loops of bowel obtaining a floppy restoration of the digestive continuity.

There is no confusion in bowel orientation, reducing the risks of swapping the intestinal tract. The double loop allows the surgeon to keep the biliary limb separate from the alimentary limb.

It is not necessary to interrupt the mesentery, reducing the risk of bleeding and in particular of internal hernias.

The surgery takes place entirely intracorporeally in a single abdominal quadrant without needing to run more than 1 docking of the robotic system, thus saving time and minimizing instruments movements. In fact, the robotic cart needs to be correctly placed, thus allowing a proper exposure of the surgical field. If this is not well planned, more than 1 docking could be required. In our technique, the reconstruction takes place only in the supramesocolic space facilitating the implementation of the intervention and reducing the risk of twisting the mesentery.

Both anastomoses are located in the sovramesocolic compartment and are very close to each other. In fact, the simple separation of the alimentary loop from the biliary limb without separating the mesentery fixes the jejunojejunostomy very close to the esophagus-jejunal anastomosis. In literature, the onset of internal hernia is rare in that area.

The safety of the procedure is confirmed by the analysis of the surgical failure made with the CUSUM chart that shows a security level maintained throughout this preliminary series.

Clinical outcomes were extremely satisfying. No patient had major complications such as bleeding or postoperative anastomotic leaks, no reoperations, or early readmission after discharge occurred.

Only 1 patient had a relevant delay in the resumption of bowel function and in starting oral intake, prolonging the hospital stay.

An interesting consideration of this preliminary series is derived from the analysis of the surgical stress (measured by the granulocytes to lymphocytes ratio).^11,23^

On the fourth postoperative day, the G:L ratio was not statistically significant in comparison to the preoperative G:L ratio.

This confirms and correlates with our clinical observation that the patient's discharge can be safely recommended from the fourth postoperative day when performing this robotic procedure, if no complications occurred.

A recent study performed by Hyun et al^11^ highlighted that there were no significant differences between the G:L ratios of robotic gastrectomy and laparoscopic gastrectomy patients, indicating that the level of surgical stress is similar for the 2 techniques. Instead, the open group of patients had a significantly greater G:L ratio.

Minimally invasive procedures, both robotic and laparoscopic surgery, allow to consider anatomical tissues also in advanced dissection and reconstruction.

This issue needs to be investigated further in a larger study, particularly, to verify any differences or possible advantages between the robotic and laparoscopic approach.

In conclusion, the double-loop method developed in our Institute was shown to be a valid simplification of the traditional techniques of reconstruction after total gastrectomy that could increase the feasibility and safety in performing a full hand-sewn intracorporeal restoration of the digestive continuity and it appears to fit the characteristics of the robotic system thus obtaining excellent postoperative clinical outcomes.

As we have reported, researchers must still explore many aspects of MIS for gastric cancer. We have a long way to go. Right now, the scientific community is wondering what strategies it should adopt in future studies.^39,40^
